# Towards a Standard Threshold for Genome Wide Significance in Dogs

**DOI:** 10.1002/age.70208

**Published:** 2026-09-12

**Authors:** Mats E. Pettersson, Katarina Tengvall, Jennifer R. S. Meadows

**Affiliations:** ^1^ Department of Medical Biochemistry and Microbiology Uppsala University Uppsala Sweden; ^2^ SciLifeLab Uppsala University Uppsala Sweden

**Keywords:** dog, GWAS, permutation, significance threshold

## Abstract

Genome‐wide association studies (GWAS) are a foundational step in tying phenotype to genotype, relying on statistical significance thresholds to distinguish true‐ from false‐positive signals of association. Dog genomics has long relied on per‐study Bonferroni thresholds of significance, basing these on SNP chip levels of markers (~100 k to > 14 M variable sites). However, as the field progresses into whole genome imputation analyses and more powerful meta‐analyses, there is a clear need to develop a standard significance threshold for common‐variant GWAS. Using 1591 dogs from the broad‐ancestry Dog10K dataset, we performed permutation analysis and developed GWAS thresholds for datasets using either 1% or 5% minor allele frequencies. The resultant *p*‐values, 4.2 × 10^−7^ and 5.0 × 10^−7^ respectively, are similar to previous Bonferroni levels (*p*‐value ~6 × 10^−7^), but less restrictive than the standard human *p*‐value, 5 × 10^−8^, which is sometimes used in dog studies. Given the diverse haplotypes from the > 320 breeds in the Dog10K input dataset, we suggest a *p*‐value of 4 × 10^−7^ as a standard significance threshold that could be applied to any dog GWAS.

## 1 Introduction

Across the field of genetics, genome‐wide association studies (GWAS) remain the classic first step in tying phenotypes to common genotypic information, that is, variant sites with a minor allele frequency (MAF) of greater than or equal to 1% or 5% in a given dataset. A NCBI PubMed search in July 2026 for ‘GWAS’, ‘dog’, ‘canine’ reveals more than 490 hits since the 2005 release of the initial dog reference genome (Lindblad‐Toh et al. 2005). This shows both the use and discussion of the utility of GWAS to investigate dog phenotypes. During the intervening 20 years, the density of markers used in dog GWAS has increased from the 50‐600 K SNP arrays, through to low‐pass sequencing followed by whole genome imputation, whole genome sequencing or a combination of technologies (e.g., Plassais et al. 2019; Drögemüller et al. 2008; Morrill et al. 2022). Studies using diverse genetic backgrounds can contain close to 15 million markers (Shan et al. 2021). Over the same time period, the threshold used to determine a likely true signal from the null hypothesis of no association has also varied, from study and marker count dependent Bonferroni thresholds, to permutation tests with false discovery rate thresholds, independent haplotype estimates and more recently, application of the human GWAS threshold (See Table 1, example *p*‐value range 2.97 × 10^−6^ to 3.45 × 10^−9^). The lack of consistent thresholding has meant that a position considered significantly associated in one study would fail to reach significance in another, without true differences in support. Although GWAS is only ever the first step in functionally tying a variant site to a phenotype, a too stringent or too relaxed threshold at this stage could gravely impact downstream analyses, and the community's common appreciation of what is significant.

**TABLE 1:** Examples of GWAS significance thresholds used in recent dog studies. Publications were selected to reflect a range of sample and marker sizes, as well as minor allele frequency inclusions.

Threshold method	Dog samples	SNP markers^a^	MAF (%)	*p‐*value^a^	Sample ancestry	References
Bonferroni	29 011	91 544	1	5.46 × 10^−7^	18 breeds	Forman et al. (2025)
	427–3093	80 686–91 269	1	5.48 × 10^−7^—6.20 × 10^−7^	Per breed for 18 breeds	Forman et al. (2025)
	91–268	14 489 548–14 984 476	1	3.34 × 10^−9^‐3.45 × 10^−9^	Multiple breeds	Shan et al. (2021)
	1187	468 649	5	2.97 × 10^−6^	One breed	Alex et al. (2025)
	470	576 754	5	8.67 × 10^−8^	Two breeds	Cook et al. (2025)
	48–307	81 230–107 878	5	4.6 × 10^−7^—6.2 × 10^−7^	Per breed for 3 breeds	Ricketts et al. (2025)
	83	2 010 300	5	2.49 × 10^−8^	One breed	Bell et al. (2023)
Human threshold	2155	8 518 951	2	5 × 10^−8^	Breed and mixed breed	Morrill et al. (2022)

Abbreviation: MAF, minor allele frequency.

^a^ If more than one experiment is reported, SNP Markers and *p*‐value summarised as range, minimum to maximum.

Here we aimed to develop a standard threshold for use in dog GWAS by using the diverse genetic variation within the Dog10K dataset, contributed by more than 320 breeds (Meadows et al. 2023). We performed permuted case–control association studies with simulated phenotypes for > 1500 dog samples and MAF of either ≥ 1% or ≥ 5%. Although these “common” allele thresholds account for both modest and ever‐increasing sample sizes in modern dog GWAS (For examples, see Table 1), we suggest that a single *p*‐value of 4 × 10^−7^ could be adopted as a standard GWAS significance threshold for the field.

## 2 Materials & Methods

Using the Dog10K (Meadows et al. 2023) variant VCF as input (https://kiddlabshare.med.umich.edu/dog10K), we used BCFtools (Li 2011) v1.19‐GCC‐13.3.0 to extract the autosomal SNP markers passing Dog10K quality control thresholds (PASS sites) for the 1591 available breed dogs (Table S1). Further filtering for polymorphic, biallelic SNPs was performed with VCFtools (Danecek et al. 2011) v0.1.16‐GCC‐13.3.0. PLINK (Chang et al. 2015) v1.9 was used to process this data for missing genotypes (‐‐geno 0.02), individual missingness (‐‐mind 0.02) and then to create two datasets based on MAF, MAF ≥ 1% and MAF ≥ 5%, and for downstream data management and association analysis. For each dataset, 10 eigenvectors (‐‐pca) were generated from linkage disequilibrium (LD) pruned data (‐‐indep‐pairwise 50 5 0.2). These vectors were subsequently used as covariates (‐‐covar) in logistic (‐‐logistic) regressions, with the association analysis procedure repeated 50 000 times using an arbitrarily assigned case or control status (‐‐fam). The phenotype status of an individual was then randomly permuted to produce a new phenotype file for each analysis (‐‐pheno). Sex was not included as a covariate as the phenotype was artificial. Rstudio v2025.05.1 + 513 and R v4.5.1 were used to permute the phenotype files, calculate lambda and collect the smallest *p*‐value from each association analysis and to subsequently visualise the results. The empirical threshold for significance for each MAF dataset was calculated using a 5% false discovery rate (FDR).

## 3 Results and Discussion

We used simulated phenotypes and a permutation protocol to calculate empirical *p*‐values from 1591 breed dogs contained within the Dog10K dataset (Meadows et al. 2023). The genetic relationships between samples and principal component variance explained across eigenvectors were illustrated in Figure S1. The GWASs used additive logistic models incorporating 10 eigenvectors as covariates, and the MAF ≥ 1% and MAF ≥ 5% datasets had 10 045 238 and 6 928 856 SNPs sites available respectively for analysis. The effective control of stratification in either GWAS permutation set was confirmed with lambda values of 1.009 ± 0.032 and 1.008 ± 0.033 for the MAF ≥ 1% and MAF ≥ 5% datasets respectively (Figure S2).

**FIGURE 1:**
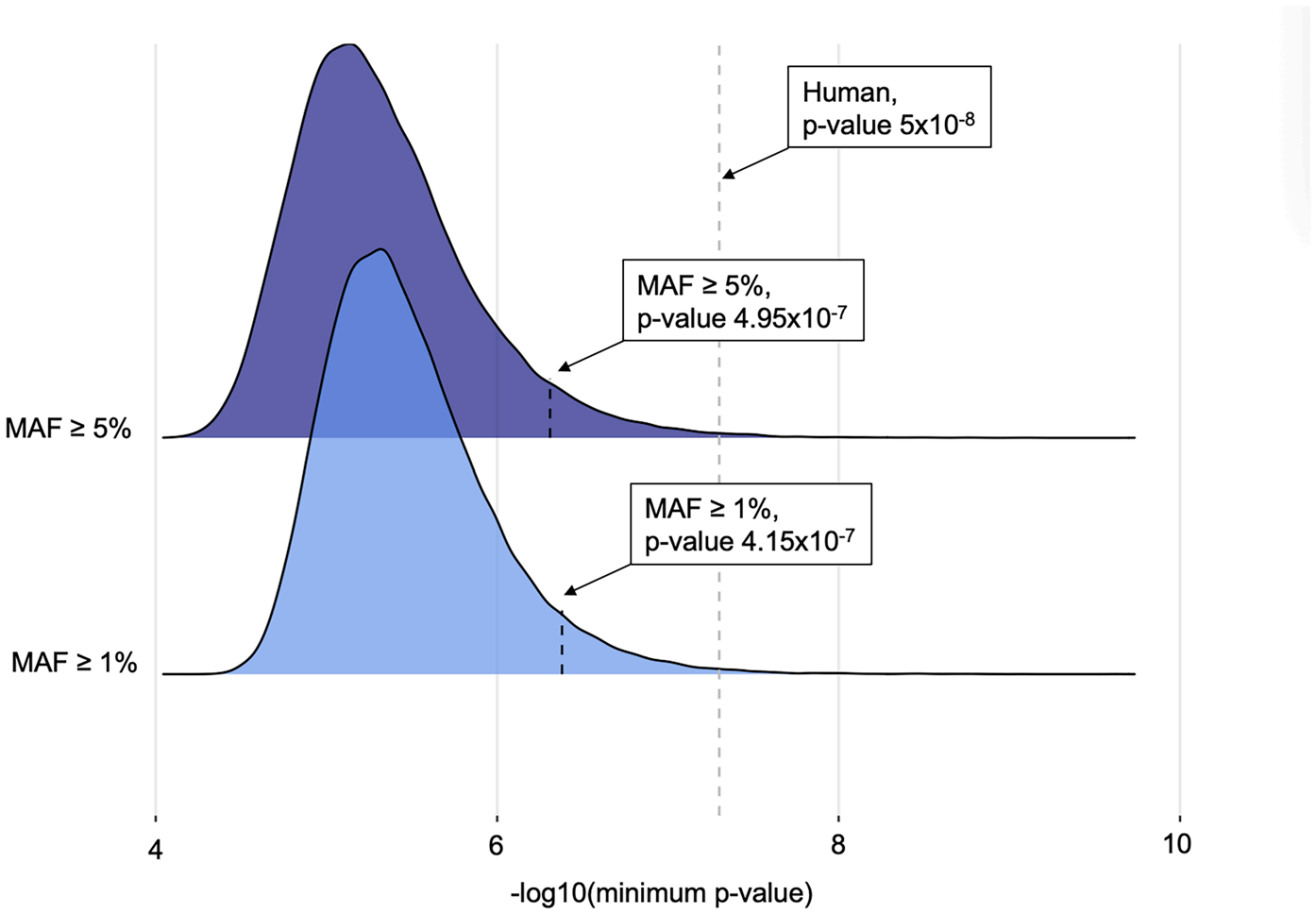
Empirical distribution of *p*‐values at MAF ≥ 1% and MAF ≥ 5%. The human genome‐wide significance value is provided for comparison.

Our threshold results of MAF ≥ 1% *p*‐value 4.2 × 10^−7^ and MAF ≥ 5% *p*‐value 5.0 × 10^−7^ (Figure 1) were similar to some of those achieved with Bonferroni methods and the 170K Illumina SNP Chip (See Table 1). The similarity of values likely reflected the original haplotype diversity contained within the whole genome sequencing pools used to develop the 170K SNP Chip (i.e., European and Asian dog breeds, plus a pool of wolves) (Vaysse et al. 2011), whereas the range of Bonferroni values was a reflection of haplotype diversity.

The dog GWAS thresholds determined here were much less stringent than the standard human *p*‐value of 5 × 10^−8^. The human threshold was estimated based on 2000 HapMap individuals, ENCODE genomics regions and a simulated case–control study permuted 10 million times (Pe'er et al. 2008). Although subsequent studies of human data have suggested that this value be adjusted for the biobank level samples and the ever reducing ‘common’ minor allele frequencies included in modern human GWASs (e.g., 13 000 to > 300 000 samples, MAF > 0.5% to 0.1% (Fadista et al. 2016; Cheruiyot et al. 2025)), to date the 5 × 10^−8^ value has stood the test of time. Nevertheless, that does not make it appropriate for use in dog studies.

There will always be a trade‐off in GWAS between the use of a less stringent GWAS threshold, and so the inclusion of more ‘significant’ results, and the need to reduce Type I and Type II errors impacting downstream analyses. Individual GWASs will be impacted by study specific factors, including population LD (e.g., longer in recently admixed or bottlenecked groups) (Kanai et al. 2016), number of individuals sampled, minor allele frequency cut‐off and GWAS model (Cheruiyot et al. 2025). All of these in combination will influence *p*‐value distribution. However, the human example shows that these factors do not negate the use of a common significance threshold for a given species.

## Author Contributions

**Mats E. Pettersson:** investigation, writing – review and editing, methodology, formal analysis. **Katarina Tengvall:** writing – review and editing, methodology. **Jennifer R. S. Meadows:** conceptualization, investigation, writing – original draft, writing – review and editing, methodology, visualization, formal analysis, resources.

## Funding

The authors have nothing to report.

## Ethics Statement

The authors have nothing to report.

## Conflicts of Interest

The authors declare no conflicts of interest.

## Supporting information


**Figure S1:** Visualisation of (A) Eigen values for MAF ≥ 1% and MAF ≥ 5%, and (B) genetic relationship of the 1591 Dog10K breed dogs using PC1 and PC2 from the MAF ≥ 5% genetic dataset. AKIT000003 = Japanese Akita, CHOW000004 = Chow Chow, BULD000002 = English Bulldog, CNTI000004 = Continental Bulldog, BHSP000005 = Bohemian Shepherd, WSSD000004 = White Swiss Shepherd Dog. For all 1591 samples used, PC1 and PC2 values, plus sample breed identifiers can be found in Table S1.
**Figure S2:** Distribution of 50 000 lambda values for (A) MAF ≥ 1% dataset and (B) and MAF ≥ 1% dataset.

**Table S1:** Sample identifiers of the 1591 breed dogs used in the GWAS threshold analysis.

## Data Availability

This manuscript used publicly available data as described in Meadows et al. (2023).

## References

[age70208-bib-0001] Alex, E. , P. Gennotte , A. Morros Nuevo , et al. 2025. “GWAS for Behavioral Traits in Golden Retrievers Identifies Genes Implicated in Human Temperament, Mental Health, and Cognition.” Proceedings of the National Academy of Sciences of the United States of America 122, no. 48: e2421757122. 41284867 10.1073/pnas.2421757122 PMC12684936

[age70208-bib-0002] Bell, S. M. , J. M. Evans , E. A. Greif , K. L. Tsai , S. G. Friedenberg , and L. A. Clark . 2023. “GWAS Using Low‐Pass Whole Genome Sequence Reveals a Novel Locus in Canine Congenital Idiopathic Megaesophagus.” Mammalian Genome: Official Journal of the International Mammalian Genome Society 34, no. 3: 464–472. 37041421 10.1007/s00335-023-09991-2 PMC10600401

[age70208-bib-0003] Chang, C. C. , C. C. Chow , L. C. Tellier , S. Vattikuti , S. M. Purcell , and J. J. Lee . 2015. “Second‐Generation PLINK: Rising to the Challenge of Larger and Richer Datasets.” GigaScience 4: 7. 25722852 10.1186/s13742-015-0047-8 PMC4342193

[age70208-bib-0004] Cheruiyot, E. K. , T. Yang , and A. F. McRae . 2025. “GWAS Significance Thresholds in Large Cohorts of European Ancestry.” Genetics 230, no. 1: iyaf056. 10.1093/genetics/iyaf056. 40146319 PMC12059634

[age70208-bib-0005] Cook, S. R. , S. Hugen , J. J. Hayward , et al. 2025. “Genomic Analyses Identify 15 Risk Loci and Reveal HDAC2, SOX2‐OT, and IGF2BP2 in a Naturally Occurring Canine Model of Gastric Cancer.” Proceedings of the National Academy of Sciences of the United States of America 122, no. 22: e2416723122. 40445765 10.1073/pnas.2416723122 PMC12146739

[age70208-bib-0006] Danecek, P. , A. Auton , G. Abecasis , et al. 2011. “The Variant Call Format and VCFtools.” Bioinformatics (Oxford, England) 27, no. 15: 2156–2158. 21653522 10.1093/bioinformatics/btr330 PMC3137218

[age70208-bib-0007] Drögemüller, C. , E. K. Karlsson , M. K. Hytönen , et al. 2008. “A Mutation in Hairless Dogs Implicates FOXI3 in Ectodermal Development.” Science (New York, N.Y.) 321, no. 5895: 1462. 18787161 10.1126/science.1162525

[age70208-bib-0008] Fadista, J. , A. K. Manning , J. C. Florez , and L. Groop . 2016. “The (In)famous GWAS P‐Value Threshold Revisited and Updated for Low‐Frequency Variants.” European Journal of Human Genetics 24, no. 8: 1202–1205. 26733288 10.1038/ejhg.2015.269 PMC4970684

[age70208-bib-0009] Forman, O. P. , J. Freyer , A. Kerr , et al. 2025. “A Splice Donor Variant in SLAMF1 Is Associated With Canine Atopic Dermatitis.” Frontiers in Veterinary Science 12: 1550617. 40612147 10.3389/fvets.2025.1550617 PMC12221898

[age70208-bib-0010] Kanai, M. , T. Tanaka , and Y. Okada . 2016. “Empirical Estimation of Genome‐Wide Significance Thresholds Based on the 1000 Genomes Project Data Set.” Journal of Human Genetics 61, no. 10: 861–866. 27305981 10.1038/jhg.2016.72 PMC5090169

[age70208-bib-0011] Li, H. 2011. “A Statistical Framework for SNP Calling, Mutation Discovery, Association Mapping and Population Genetical Parameter Estimation From Sequencing Data.” Bioinformatics 27, no. 21: 2987–2993. 21903627 10.1093/bioinformatics/btr509 PMC3198575

[age70208-bib-0012] Lindblad‐Toh, K. , C. M. Wade , T. S. Mikkelsen , et al. 2005. “Genome Sequence, Comparative Analysis and Haplotype Structure of the Domestic Dog.” Nature 438, no. 7069: 803–819. 16341006 10.1038/nature04338

[age70208-bib-0013] Meadows, J. R. S. , J. M. Kidd , G.‐D. Wang , et al. 2023. “Genome Sequencing of 2000 Canids by the Dog10K Consortium Advances the Understanding of Demography, Genome Function and Architecture.” Genome Biology 24, no. 1: 187. 37582787 10.1186/s13059-023-03023-7 PMC10426128

[age70208-bib-0014] Morrill, K. , J. Hekman , X. Li , et al. 2022. “Ancestry‐Inclusive Dog Genomics Challenges Popular Breed Stereotypes.” Science (New York, N.Y.) 376, no. 6592: eabk0639. 35482869 10.1126/science.abk0639 PMC9675396

[age70208-bib-0015] Pe'er, I. , R. Yelensky , D. Altshuler , and M. J. Daly . 2008. “Estimation of the Multiple Testing Burden for Genomewide Association Studies of Nearly All Common Variants.” Genetic Epidemiology 32, no. 4: 381–385. 18348202 10.1002/gepi.20303

[age70208-bib-0016] Plassais, J. , J. Kim , B. W. Davis , et al. 2019. “Whole Genome Sequencing of Canids Reveals Genomic Regions Under Selection and Variants Influencing Morphology.” Nature Communications 10, no. 1: 1489. 10.1038/s41467-019-09373-w PMC6445083 30940804

[age70208-bib-0017] Ricketts, S. L. , S. Ahonen , L. Pettitt , et al. 2025. “Common Variants in the CPT1A Gene Are Associated With Cataracts in Northern Breeds of Domestic Dog.” PLoS One 20, no. 4: e0320878. 40184359 10.1371/journal.pone.0320878 PMC11970653

[age70208-bib-0018] Shan, S. , F. Xu , and B. Brenig . 2021. “Genome‐Wide Association Studies Reveal Neurological Genes for Dog Herding, Predation, Temperament, and Trainability Traits.” Frontiers in Veterinary Science 8: 693290. 34368281 10.3389/fvets.2021.693290 PMC8335642

[age70208-bib-0019] Vaysse, A. , A. Ratnakumar , T. Derrien , et al. 2011. “Identification of Genomic Regions Associated With Phenotypic Variation Between Dog Breeds Using Selection Mapping.” PLoS Genetics 7, no. 10: e1002316. 22022279 10.1371/journal.pgen.1002316 PMC3192833

