# Supplementary material for: Towards a Standard Threshold for Genome Wide Significance in Dogs
Source: Anim Genet. 2026 Sep 12;57(5):e70208. doi: 10.1002/age.70208 (PMC13570322; doi:10.1002/age.70208)
Supplement: Supplementary file 1 [file AGE-57-0-s002.docx]

**Fig. S1**. Visualisation of (**A**) Eigen values for MAF ≥ 1% and MAF ≥ 5%, and (**B**) genetic relationship of the 1,591 Dog10K breed dogs using PC1 and PC2 from the MAF ≥ 5% genetic dataset. AKIT000003 = Japanese Akita, CHOW000004 = Chow Chow, BULD000002 = English Bulldog, CNTI000004 = Continental Bulldog, BHSP000005 = Bohemian Shepherd, WSSD000004 = White Swiss Shepherd Dog. For all 1,591 samples used, PC1 and PC2 values, plus sample breed identifiers can be found in Table S1.

**Fig. S2**. Distribution of 50,000 lambda values for (**A**) MAF ≥ 1% dataset and (**B**) and MAF ≥ 1% dataset.
